# Items of General Interest

**Published:** 1916-04

**Authors:** 


### Items of General Interest.

The Texas Roentgen Ray Society will hold its next meeting in Galveston May 8th from 1 to 11 p. m.

We had a splendid program at our last meeting and expect a better one this time. All doctors interested in this line of work are cordially invited to attend.

Remember the date, Monday, a day in advance of the State Association.

DR. J. M. MARTIN,
President.
DR. J. W. TORBETT,
Secretary and Treasurer.

Dr. Arthur M. McElhannon, of Sherman, has been appointed by Governor Ferguson as a member of the State Board of Medical Examiners, succeeding his brother, who recently resigned.

The plant of the Chattanooga Aseptic Cotton Company was partially destroyed by fire recently. It has been operating night and day for several months on orders for medicated cotton for the European allies. The loss is estimated at $65,000.

---

As a result of an extensive health campaign conducted in Texas by the International Health Association, a part of the Rockefeller Foundation, co-operating with the State Health Department, State Health Officer W. B. Collins has announced that a number of counties in the southeastern part of Texas have made an appropriation of $800 each to aid in the campaign. This is supplemented by a like amount by the campaign.

---

A free medical dispensary has been opened in a room of the new courthouse at Abilene. It is in charge of charitable women of the town and its purpose is to provide means by which persons or families who are not able to employ physicians may be treated free of cost.

---

Dr. Kilgore, a well known physician of Germantown, was attacked and badly beaten by a farmer recently, the trouble arising over the loss of some poultry on the farm. The wounded man was carried to Goliad for treatment, and officers placed the farmer in jail.

---

Dr. J. D. Spear, of Dayton, was recently appointed county health officer of Liberty county by Judge C. N. Smith.

---

The city officials of Matamoras, with Mayor Salvador Cardenas, who was appointed head of the local administration, are to conduct a vigorous fight against smallpox in that city. Three physicians of that city have been appointed to look after sanitary conditions in the Mexican city, and will conduct a house to house campaign.

---

The mosquito is the most hated citizen of Brazil. The latest charge lodged against him is that of carrying and transmitting leprosy. It is estimated that leprosy, in its various forms, has about 26,000 victims in Brazil.

---

It is believed by local physicians that in the event of an extended campaign in Mexico a special camp may be established at Bay City for the treatment of typhus fever.

---

As a result of recent tests made by Dr. A. F. Beverly in cooperation with the State Board of Health, it has been found that

26 per cent of the students from East Texas at the Deaf and Dumb Institute are infected with hookworm.

---

According to Dr. C. C. Pierce, senior surgeon of the United States Public Health Service, the United States have more to fear from disease than from Villa bullets. Dr. Pierce says the greatest sources of danger to the troops are from typhus-carrying lice and malarial fever, which are quite common in the interior of Mexico.

---

The Chicago Medical Society has suspended Dr. J. H. Haiselden from membership for "conduct unbecoming the ethics of the profession." The action was a result of the famous Baby Bollinger case.

---

Dr. O. J. Cook, who for some time has been serving as city health officer for Laredo, is now with General Pershing in Mexico.

---

The *American Journal of Gastro-Enterology* has combined with the *Proctologist,* and hereafter will be published (beginning with the March number, first of the year) as the *Proctologist and Gastro-enterologist,* from St. Louis. Dr. Lewis Brinton, Philadelphia, and Dr. Anthony Bassler, New York, will have editorial charge of the gastro-enterology; Dr. A. L. Benedict, Buffalo, editor of dietetics; Dr. Rollin H. Barnes, St. Louis, will be managing editor and publisher.

---

Dr. Felix Mendel, of Germany, has satisfactorily demonstrated to the medical fraternity of that country that one of the most effectual remedies for wounds is found in a powder, consisting in a mixture of ten parts of bicarbonate of soda, nine parts acetic acid (vinegar), and nineteen parts sugar. Superficial wounds are covered with a thick layer of the powder, but deeper wounds are completely filled with it. As soon as the mixture is placed on a raw sore carbon dioxide is liberated, and this causes a constant flow of fluid from the wound. Inflammation is quickly checked by using this fluid.

---

The State Medical Society will, it is stated, take steps looking to action in the courts to deprive of license to practice medicine forty or fifty physicians who have illegally prescribed habit-forming drugs and have been convicted therefor. A conviction of such an act should carry the cancellation of license with it.

The United States Public Health Service brands strong drink as the most efficient ally of pneumonia. It declares that alcohol is the handmaiden of the disease which produces 10 per cent of the deaths in the United States.

---

Dr. John A. Snell of Soochow, China, who has been a surgeon in the hospital there for the last seven years under the direction of the foreign missions board of the Southern Methodist Episcopal Church, is in Galveston for a visit to the State Medical College. Dr. Snell is on a one year's leave of absence for the purpose of doing post-graduate work in New York City and of visiting medical colleges in the United States in the interest of medical work in China.

---

Doctor—"That blonde they've just brought into the hospital ward is light-headed."

Nurse—"Law, doctor, so is any blonde."—*Rice Rustler.*

---

In a prosperous rural community the village doctor was also the superintendent of the Sunday school. Incidentally he taught a class of small boys.

"Willie," impressively remarked the doctor one Sunday morning to a bright faced youngster, "can you tell me what must we do in order to get to heaven?"

"Yes, sir," was the prompt response of Willie; "we must die."

"That is very true," said the doctor encouragingly, "but can't you tell me what we must do before we die?"

"Yes, sir," was the startling rejoinder of little Willie, "we must get sick and send for you."—*Daily Panhandle.*

